# Controlling Nutritional Status Scores Predict Postoperative Acute Kidney Injury in Living Donor Liver Transplantation

**DOI:** 10.1111/ctr.70562

**Published:** 2026-05-14

**Authors:** Akira Katayama, Ezeldeen Abuelkasem, Marianne M. Ligon, David W. Wang

**Affiliations:** ^1^ Department of Anesthesiology and Perioperative Medicine University of Pittsburgh Pittsburgh, PA USA; ^2^ Department of Anesthesiology and Resuscitology Okayama University Graduate School of Medicine Dentistry and Pharmaceutical Sciences Okayama Japan

**Keywords:** acute kidney injury, CONUT score, living donor liver transplantation, nutritional assessment

## Abstract

**Background:**

Acute kidney injury (AKI) is a frequent complication after liver transplantation (LT), and malnutrition is a known risk factor. The Controlling Nutritional Status (CONUT) score, based on serum albumin, lymphocyte count, and cholesterol, has been associated with postoperative AKI in other surgeries, but its relevance to LT remains unclear. This study aimed to evaluate the utility of the CONUT score for predicting AKI in living donor LT (LDLT) recipients.

**Methods:**

We retrospectively analyzed 480 patients who underwent LDLT from January 2012 to June 2023. The CONUT score was calculated using preoperative laboratory data. The primary outcome was AKI incidence, defined by KDIGO criteria, and compared across CONUT grades. Multivariate logistic regression was used to identify independent predictors of AKI. Secondary outcomes included early allograft dysfunction (EAD), hospital length of stay (LOS), 30‐day and 1‐year mortality, and postoperative model for end‐stage liver disease (MELD) scores on days 1, 3, 5, and 7.

**Results:**

Of the 480 patients, 150 (31.3%) developed AKI. AKI incidence significantly differed by CONUT grade (*p* = 0.003). Higher CONUT grades were associated with longer ventilation time (*p* = 0.008), prolonged hospital LOS (*p* = 0.001), more frequent EAD (*p* < 0.001), and higher postoperative MELD scores (*p* = 0.01). The CONUT score was independently associated with AKI in multivariate analysis (OR 1.14, 95% CI 1.05–1.24, *p* = 0.001).

**Conclusion:**

The CONUT score independently predicts postoperative AKI in LDLT and may be a useful tool for preoperative risk stratification and perioperative management.

**Abbreviations**
AKI: acute kidney injury
AUC: area under the curve
BMI: body mass index
CI: confidence interval
CIT: cold ischemic time
CONUT: controlling nutritional status
DM: diabetes mellitus
EAD: early allograft dysfunction
GRWR: graft‐to‐recipient weight ratio
ICU: intensive care unit
IL: interleukin; INR, international normalized ratio
IQR: interquartile range
KDIGO: kidney disease improving global outcomes
LDLT: living donor liver transplantation
LOS: length of stay
LT: liver transplantation
MELD: model for end‐stage liver disease
NRI: nutritional risk index
OR: odds ratio
PNI: prognostic nutritional index
POD: postoperative day
ROC: receiver operating characteristics
TNF: tissue necrosis factor
WIT: warm ischemic time

## 1 Introduction

Acute kidney injury (AKI) is a common complication following liver transplantation (LT) with a reported incidence ranging from 31.8% to 63.1% [1, 2]. AKI is associated with prolonged intensive care unit (ICU) length of stay (LOS), total hospital LOS, postoperative ventilation time, decreased graft survival, and higher mortality [2, 3, 4]. The etiology of post‐LT AKI is typically considered multifactorial, including factors related to recipients, donors, intraoperative events, and early management after LT [5]. Malnutrition, a common feature in end‐stage liver disease patients, is also a known risk factor for postoperative AKI [6]. Patients with end‐stage liver disease are malnourished due to inadequate dietary intake of nutrients, reduced synthesis or absorption of protein, increased protein loss, disturbances in substrate utilization, and increased levels of pro‐inflammatory cytokines resulting in a hypermetabolic state [7]. Preoperative evaluation for nutritional status of LT recipients may have a significant impact on post‐LT outcomes. Moreover, nutritional status is one of the few modifiable preoperative factors, and identifying at‐risk patients may provide an opportunity for targeted intervention to improve postoperative outcomes such as AKI. Therefore, incorporating objective nutritional assessments into preoperative evaluation may be clinically meaningful in optimizing perioperative management strategies for LT recipients.

The Controlling Nutritional Status (CONUT) score is a laboratory‐based nutritional index derived from serum albumin, total lymphocyte count, and total cholesterol level. Originally developed as a screening tool for hospitalized patients [8], it offers a practical and objective alternative to other established nutritional or functional indices such as sarcopenia, handgrip strength, or frailty, which often require specialized equipment or subjective assessment. Because it relies solely on routine laboratory data, the CONUT score is readily applicable in the preoperative setting for risk stratification. The CONUT score has been widely validated as a prognostic marker for postoperative mortality in several surgical settings, including colorectal cancer, gastric cancer, esophageal carcinoma, hepatocellular carcinoma, and LT [9, 10, 11, 12, 13, 14]. Furthermore, it has been demonstrated that the CONUT score is associated with postoperative AKI in patients undergoing cardiac surgery and major abdominal surgery [15, 16]. However, the predictive value of the CONUT score for postoperative AKI in LT has not yet been investigated.

This study aimed to evaluate the CONUT score as a predictive value for the incidence of AKI after living donor LT (LDLT).

## 2 Methods

We retrospectively reviewed the medical records of patients who underwent LDLT between January 2012 and June 2023 at University of Pittsburgh Medical Center (Pittsburgh, USA). All patients who underwent LDLT were enrolled in this study. Patients aged < 18 years and those with chronic kidney disease dependent on hemodialysis were excluded. Patients with insufficient data to calculate the CONUT score within 3 months before LDLT were also excluded from this study. This study was approved by the University of Pittsburgh institutional review board (STUDY20050148) and conducted in accordance with both the Declarations of Helsinki and Istanbul. The requirement for written informed consent was waived by the institutional review board owing to the retrospective design of the study.

### 2.1 Data Collection

We investigated perioperative information, including preoperative characteristics, intraoperative variables, and postoperative courses. Demographics and clinical data were extracted from the electronic medical records. Preoperative data included sex, age, body mass index (BMI), etiology of liver failure, hypertension, diabetes mellitus (DM), model for end‐stage liver disease (MELD) score, and laboratory findings evaluated within 1 week before surgery. We collected intraoperative data including duration of procedure, blood loss, and volume of infusion from anesthetic records. Donor information including age, graft weight, graft‐to‐recipient weight ratio (GRWR), cold ischemic time (CIT), and warm ischemic time (WIT) were also retrieved from electronic medical records. The CONUT score was calculated based on serum albumin levels, total lymphocyte counts, and total cholesterol levels, and each finding was measured within 3 months before LT (Table 1). Postoperative data included the incidence of AKI and early allograft dysfunction (EAD), ICU and hospital LOS, ventilation time, mortality at 30‐days and 1‐year, and MELD score on postoperative days (POD) 1, 3, 5, and 7. AKI was defined according to the Kidney Disease Improving Global Outcomes (KDIGO) definition [17]: an increase in serum creatinine of 0.3 mg/dL within 48 h or a rise 1.5 times baseline or more within 7 days. We set the baseline creatinine as the most recent serum creatinine level within 7 days prior to surgery. EAD was defined as the presence of one or more of the following variables: bilirubin ≥ 10 mg/dL on POD 7, or INR ≥ 1.6 on POD 7 according to the Olthoff criteria [18].

**TABLE 1:** The CONUT score.

Parameter	Malnutrition			
	Normal	Light	Moderate	Severe
Serum albumin, g/dL score	≥ 3.5 0	3.0–3.49 2	2.5–2.99 4	< 2.5 6
Total lymphocyte, /µL score	≥ 1600 0	1200–1599 1	800–1199 2	< 800 3
Total cholesterol, mg/dL score	≥ 180 0	140–179 1	100–139 2	< 100 3
CONUT score	0–1	2–4	5–8	9–12
CONUT score = Albumin score + Lymphocyte score + Cholesterol score				

Abbreviation: CONUT, controlling nutritional status.

### 2.2 Outcomes

The primary outcome was the incidence of postoperative AKI. Patients were allocated based on their primary outcome incidence into non‐AKI and AKI groups. Patient characteristics and intraoperative variables were compared between the groups. Secondary outcomes included the incidence of EAD, ICU and hospital LOS, mortality at 30‐days and 1‐year, and MELD scores on POD 1, 3, 5, and 7. We compared the differences of primary and secondary outcomes among the CONUT grades (Normal, Light, Moderate, and Severe).

### 2.3 Statistical Analysis

Continuous variables are expressed as mean ± standard deviation or median with interquartile range (IQR), depending on the data distribution. Categorical data are reported as absolute number with percentages (%). Differences between groups were assessed using the Mann‐Whitney U test for continuous variables and Chi‐square test for categorical variables. For primary and secondary outcomes, in order to identify the differences among the CONUT grades, Kruskal‐Wallis test was applied for continuous variables and Chi‐square test was used for categorical variables. To investigate the impact of prognostic factors associated with the incidence of AKI, a logistic regression model was used for multivariate analysis. Multivariate analysis was performed with known risk factors for post‐LT AKI, including age, sex, BMI, DM, MELD score, CIT, GRWR, and intraoperative blood loss [19, 20, 21]. Odds ratios (ORs) and 95% confidence intervals (CIs) were calculated.

Receiver operating characteristic (ROC) curve was constructed to evaluate the CONUT score as a predictor of AKI, and the area under the curve (AUC) was also analyzed. The optimal cut‐off point was identified using the Youden index. A p value < 0.05 was considered statistically significant. All analyses were conducted using StataSE version.17.0 (College Station, TX) and EZR (Saitama Medical Center, Jichi Medical University, Saitama, Japan), a graphical user interface for R version 4.4.1 (R Foundation for statistical Computing, Vienna, Austria).

## 3 Results

The patient flow chart is shown in Figure S1. A total of 480 patients were included for analysis. Among all eligible patients, median CONUT score was 5 (IQR: 3–7) (Figure 1A). According to the CONUT grading system (Table 1), 37 patients (7.7%) were considered Normal, 149 patients (31.0%) were Light, 223 patients (46.5%) were Moderate, and 71 patients (14.8%) were Severe (Figure 1B). Patient characteristics are summarized in Table 2. Among all eligible patients, 150 (31.3%) developed AKI according to the KDIGO criteria. Of these, 116 (24.2%) had stage 1, 28 (5.8%) had stage 2, and 6 (1.3%) had stage 3 AKI. As shown in Table 2, patients with AKI were more likely to have higher BMI, MELD score, bilirubin level, and international normalize ratio (INR) (*p* = 0.004, *p*<0.001, *p*<0.001, and *p*<0.001, respectively). They also had lower serum albumin level and platelet counts (*p*<0.001 and *p* = 0.002, respectively). The CONUT score was significantly higher in those that developed AKI compared to those that did not develop AKI (*p*<0.001). Table 2 also shows the intraoperative variables compared between the groups. Patients with AKI were more likely to have a longer procedure duration, lower GRWR, lower administration of colloid, and lower urinary output (*p* = 0.04, *p* = 0.003, *p* = 0.02, and *p*<0.001, respectively). Blood loss was similar among the groups (*p* = 0.07).

**TABLE 2:** Preoperative and intraoperative characteristics.

	Non‐AKI (*n* = 330)	AKI (*n* = 150)	*p* value
Age, years	59 [50, 66]	58 [50, 65]	0.27
Sex (Female), *n* (%)	129 (39.1%)	54 (36.0%)	0.52
BMI, kg/m2	27.7 [24.9, 32.2]	29.5 [26, 34.4]	0.004
Etiology			0.01
NASH, *n* (%)	96 (29.1%)	50 (33.3%)	
Alcoholic, *n* (%)	54 (16.4%)	29 (19.3%)	
Malignancy, *n* (%)	61 (18.5%)	20 (13.3%)	
Hypertension, *n* (%)	164 (49.7%)	76 (50.7%)	0.84
DM, *n* (%)	128 (38.8%)	54 (36.0%)	0.84
CONUT score	5 [2, 6]	6 [3, 7]	<0.001
MELD score	15 [9, 19]	18 [12, 20]	<0.001
Donor age, years	37 [29, 46]	38 [27, 45]	0.27
AST, U/L	44 [30, 68]	45.5 [31, 65]	0.57
ALT, U/L	30 [18, 49]	29 [18, 45]	0.55
Albumin, g/dL	3.4 [3, 3.9]	3.1 [2.8, 3.5]	<0.001
Bilirubin, mg/dL	1.9 [1.1, 3.4]	2.9 [1.7, 4.8]	<0.001
Platelet counts, ×103/µL	102 [67, 148]	81 [60, 120]	0.002
INR	1.5 [1.3, 1.7]	1.6 [1.4, 1.9]	<0.001
Creatinine, mg/dL	0.9 [0.7, 1.2]	0.9 [0.7, 1.2]	0.34
Sodium, mEq/L	136 [133, 139]	136 [133, 139]	0.86
CIT, min	111 [95, 127]	114 [99, 133]	0.09
WIT, min	25 [21, 29]	25 [22, 29]	0.87
Duration of procedure, min	543 [483, 601]	560 [490, 654]	0.04
Graft weight, g	900 [780, 1008]	870 [760, 970]	0.22
GRWR	1.06 [0.86, 1.28]	0.98 [0.85, 1.12]	0.003
Crystalloid, mL	6662 [5186, 8243]	6878 [5618, 9351]	0.02
Colloid, mL	1500 [750, 2000]	1125 [500, 2000]	0.02
RBC, mL	0 [0, 600]	125 [0, 900]	0.16
Plasma, mL	0 [0, 0]	0 [0, 0]	0.28
Platelet, mL	0 [0, 0]	0 [0, 0]	0.21
Blood loss, mL	625 [500, 1000]	750 [500, 1200]	0.07
Urinary output, mL	1375 [790, 2000]	1015 [690, 1585]	<0.001

Abbreviations: AKI, acute kidney injury; BMI, body mass index; CIT, cold ischemic time; CONUT, Controlling Nutritional Status; DM, diabetes mellitus; GRWR, graft‐to‐recipient weight ratio; INR, international normalized ratio; MELD, model for end‐stage liver disease; NASH, non‐alcoholic steatohepatitis; RBC, red blood cell; WIT, warm ischemic time.

**FIGURE 2:**
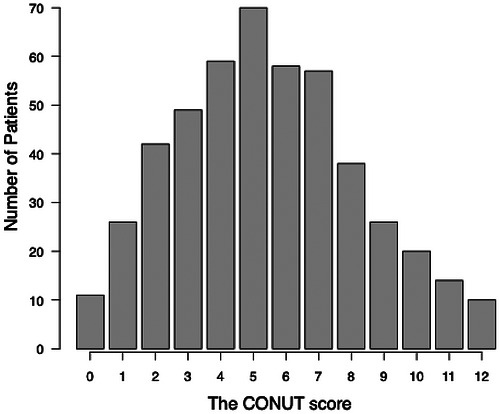
The incidence of AKI by the CONUT grade. *p* value is < 0.008 for Bonferroni significance in this analysis. AKI, acute kidney injury; CONUT, controlling nutritional status.

Figure 2 shows the primary outcome. The incidence of AKI was significantly different among the CONUT grades (Normal: 5/37 (13.5%), Light: 38/149 (25.5%), Moderate: 76/223 (34.1%), Severe: 31/71 (43.7%), *p* = 0.003). The pairwise comparison showed a significant difference between the grade of Normal and Severe (*p* = 0.003). To identify the prognostic factors closely correlated with the incidence of AKI, multivariate analyses were applied (Table 3). In multivariate analysis, the CONUT score and intraoperative blood loss were independent prognostic factors for the development of AKI (OR 1.14, 95% CI: 1.05‐1.24, *p* = 0.001 and OR 1.27, 95% CI: 1.01‐1.61, *p* = 0.04, respectively). In ROC analysis, the optimal cut‐off value of the CONUT score for predicting the incidence of AKI was 7, with a sensitivity of 48.0%, specificity of 71.8%, and AUC of 0.62 (95% CI 0.57–0.68) (Figure S2). In stratified analyses, the association between the CONUT score and postoperative AKI was more pronounced in patients with higher MELD scores. Among recipients with MELD score ≥20, the CONUT score remained independently associated with AKI (OR 1.26, 95% CI: 1.09‐1.46, *p* = 0.002), whereas no significant association was observed in those with MELD score <20 (OR 1.04, 95% CI: 0.93‐1.16, *p* = 0.50). Although the interaction between CONUT score and MELD category did not reach statistical significance (*p* for interaction = 0.09), the discriminative performance of the CONUT score improved in the higher MELD subgroup (AUC 0.68). In an additional exploratory analysis using a higher cutoff (MELD score ≥25), the association between the CONUT score and postoperative AKI appeared further accentuated (OR 1.36, 95% CI: 1.05‐1.77, *p* = 0.02), with a corresponding increase in predictive ability (AUC 0.70). However, this subgroup included only 57 patients, and these findings should therefore be interpreted with caution.

**FIGURE 1A:**
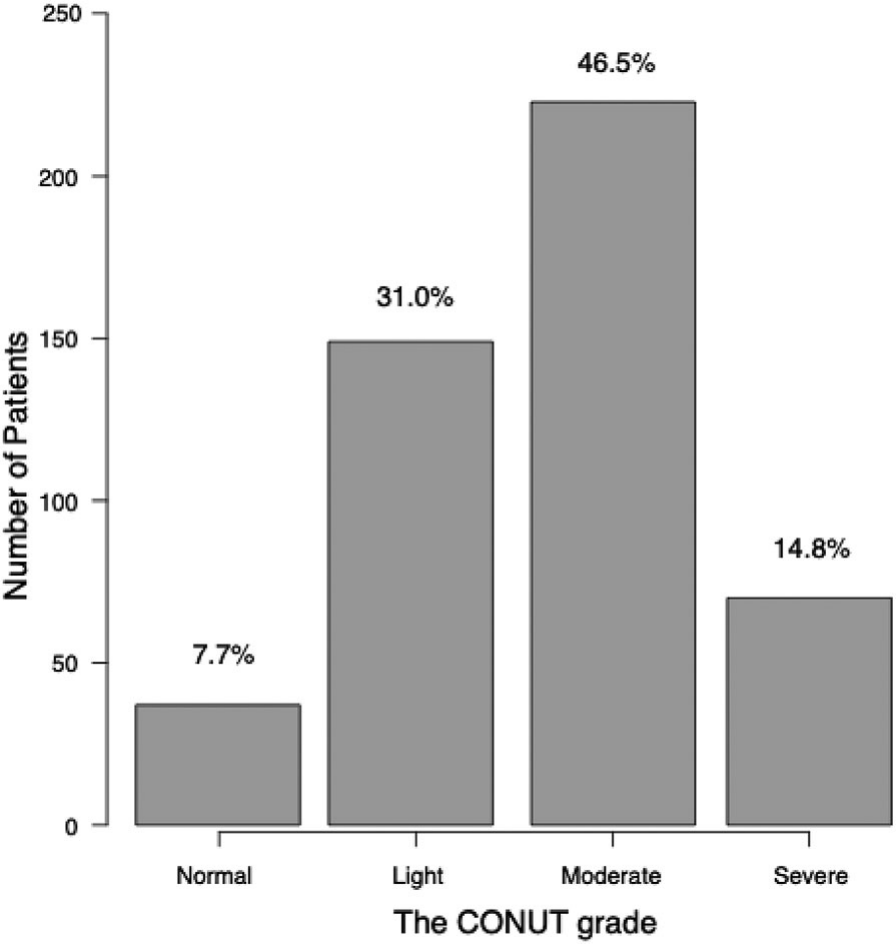
The distribution of the CONUT score. CONUT, controlling nutritional status.

**FIGURE 1B:**
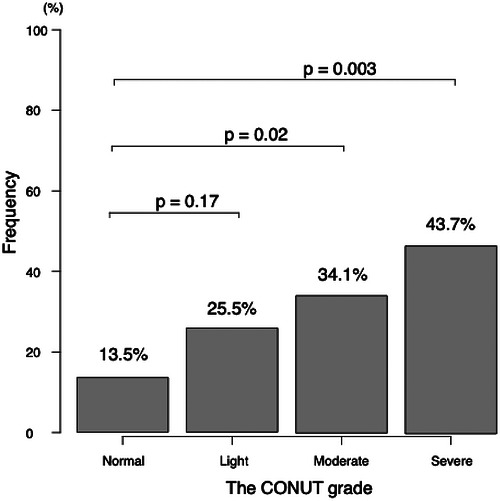
The distribution of the CONUT grade CONUT, controlling nutritional status.

**TABLE 3:** Multivariate logistic regression analysis for post‐LT AKI.

	Odds ratio	95% CI	*p* value
Age, years (every 10 years increase)	0.94	0.78–1.14	0.54
Sex (male)	0.93	0.60–1.46	0.76
BMI: Obesity (Obesity: BMI ≥30 kg/m^2^)	1.46	0.93–2.32	0.10
DM type II	0.75	0.47–1.21	0.24
MELD score	1.01	0.98–1.05	0.44
CIT, min	1.00	0.99–1.01	0.23
GRWR	0.72	0.41–1.27	0.26
Blood loss, L	1.27	1.01–1.61	0.04
CONUT score (continuous variables)	1.14	1.05–1.24	0.001

Abbreviations: AKI, acute kidney injury; BMI, body mass index; CIT, cold ischemic time; CONUT, controlling nutritional status; DM, diabetes mellitus; GRWR, graft‐to‐recipient weight ratio; LT, liver transplantation; MELD, model for end‐stage liver disease.

In a supplementary analysis, serum albumin alone was also found to be an independent predictor of postoperative AKI (OR 0.55, 95% CI: 0.39–0.79, *p* = 0.001), whereas total lymphocyte count (OR 0.98 per 100/µL increase, 95% CI: 0.95‐1.02, *p* = 0.36) and total cholesterol (OR 0.97 per 10 mg/dL increase, 95% CI: 0.94‐1.01, *p* = 0.13) were not significantly associated with AKI. When both CONUT score and albumin were included in the same model, neither remained statistically significant (*p* = 0.26 and *p* = 0.19, respectively), likely due to multicollinearity. The ROC analysis showed no significant difference in AUC between CONUT score and albumin (*p* = 0.47).

Secondary outcomes are shown in Table 4 and Figure S3. The ventilation time, incidence of EAD, hospital LOS, and postoperative MELD score were significantly different among the CONUT grades (*p*<0.001, *p* = 0.008, *p* = 0.001, and *p* = 0.01, respectively). However, ICU LOS, and mortality at 30‐days and 1‐year were similar among the groups (*p* = 0.12, *p* = 0.22, and *p* = 0.76, respectively).

**TABLE 4:** Secondary outcomes.

	CONUT grade				*p* value
	Normal	Light	Moderate	Severe	
Ventilation time, hr.	0 [0, 5]	0 [0, 12]	4 [0, 13]	9 [0, 14]	0.008
EAD, *n* (%)	3 (8.1%)	24 (15.9%)	58 (26.5%)	27 (37.0%)	<0.001
ICU LOS, day	2 [1, 1]	2 [1, 2]	2 [1, 2]	2 [1, 3]	0.12
Hospital LOS, day	7 [5, 9]	7 [5, 10]	9 [6, 16]	11 [6, 18]	0.001
30‐days mortality, *n* (%)	1 (2.7%)	3 (2.0%)	7 (3.1%)	0 (0%)	0.48
1‐year mortality, *n* (%)	2 (5.4%)	13 (8.6%)	18 (8.1%)	6 (8.5%)	0.93

Abbreviations: CONUT, controlling nutritional status; EAD, early allograft dysfunction; ICU, intensive care unit; LOS, length of stay.

## 4 Discussion

In this retrospective review, the incidence of AKI after LT was significantly different among the CONUT grades. Multivariate analysis revealed that poor nutritional status, as evaluated by the CONUT score, was an independent risk factor for the incidence of AKI. Furthermore, the incidence of EAD, ventilation time, hospital LOS, and postoperative MELD scores appeared to vary across CONUT grades in our cohort. To the best of our knowledge, this study is the first to demonstrate the correlation between the CONUT score and postoperative AKI after LT. While the CONUT score was independently associated with postoperative AKI, its discriminative performance was modest, indicating that it may be best applied as a complementary rather than definitive risk stratification tool.

Nutritional assessment is pivotal for patients with end‐stage liver disease. Malnutrition is frequently found in this population and can significantly influence their prognosis [22]. In fact, although only a small proportion of patients in this cohort were classified as having a normal CONUT score, this likely reflects the underlying nutritional vulnerability common in patients with end‐stage liver disease. The value of the CONUT score lies not in dichotomizing patients into normal or abnormal, but in stratifying nutritional risk and identifying those who may benefit from targeted preoperative optimization. Although no literature has identified the relationship between CONUT score and post‐LT AKI, the CONUT score has been reported to be independently associated with postoperative AKI in other surgical procedures, including cardiac surgery and major abdominal surgery. Usta and Engin [15] revealed in a retrospective study including 439 patients who underwent cardiac surgery with cardiopulmonary bypass that the CONUT score calculated in the preoperative period was an independent predictor of the development of AKI. In another study performed on 2775 patients who underwent major abdominal surgery [16], poor preoperative nutritional status assessed by CONUT score was independently associated with an elevated risk of AKI. Our finding of the prognostic significance of CONUT score for postoperative AKI in LDLT was consistent with these studies. Furthermore, our finding that the incidence of AKI increased progressively with higher CONUT grades suggests that improving preoperative nutritional status may have the potential to reduce the risk of postoperative AKI, underscoring the clinical utility of the CONUT score as a modifiable risk indicator. Even if nutritional status cannot be normalized, partial improvement in the CONUT score may still contribute to reducing the risk of postoperative AKI. The CONUT score demonstrated limited to moderate discriminative ability in our cohort and therefore should not be regarded as a stand‐alone predictive tool. Rather, its clinical value may lie in its simplicity, objectivity, and ability to provide complementary information when integrated into a broader perioperative risk assessment framework. In this context, stratified analyses suggested that the modest overall predictive performance of the CONUT score may partly reflect the relatively low‐risk profile of the overall cohort. Specifically, the association between the CONUT score and postoperative AKI appeared more pronounced in patients with higher MELD scores, with improved discriminative performance observed in higher MELD subgroups.

The mechanisms of how malnutrition contributes to the development of AKI have not yet been totally elucidated, and explanations underlying the relationship between nutritional status and AKI have been suggested. Malnutrition is known to be associated with increased inflammatory cytokines, including interleukin (IL)‐1, ‐6 and tumor necrosis factor (TNF)‐a. These inflammatory cytokines exacerbate vasoconstriction and oxidative stress, resulting in renal injury [23]. In addition, protein‐calorie malnutrition induces significant alterations in renal hemodynamics, renal concentration capacity, and renal acid excretion. Such alterations lead to decreased glomerular filtration rate, reduced renal blood flow, and decreased acid load excretion, leading to the development of AKI [24]. Albumin, one of the components of the CONUT score, plays a vital role in preserving renal function. Albumin has a renal protective effect by improving renal perfusion, inhibiting apoptosis of renal tubular cells, and promoting the proliferation of renal tubular cells [20, 25]. Thus, a lower albumin level has been identified as an independent risk factor for AKI in various surgical procedures including LT [6, 26]. Lymphocytes, important cells of the immune system, are influenced by malnutrition in reducing their functional capacity [27]. It has been demonstrated that lymphocytes play a key role in the initiation, propagation, and recovery phase of AKI [28], and that preoperative low lymphocyte counts are significantly associated with the incidence of postoperative AKI [27, 29]. Total cholesterol is also included in the CONUT score calculation. Multiple studies have identified the correlation between cholesterol and postoperative AKI [30, 31], though the mechanisms for this remain unclear. In our supplementary analysis, among the three components of the CONUT score, only serum albumin was independently associated with postoperative AKI, while total lymphocyte count and total cholesterol were not. This finding supports the notion that albumin is the central determinant of the CONUT score's predictive ability. However, the CONUT score incorporates additional immunologic and metabolic domains through lymphocyte count and cholesterol, providing a more comprehensive assessment of nutritional status. Its simplicity and reliance on routinely available laboratory data make it a practical and objective tool for clinical risk stratification compared to using albumin alone.

In the present study, intraoperative blood loss was also independently associated with post‐LT AKI. Blood loss reduces blood flow to the kidneys, interfering with their ability to filter blood. In fact, multiple studies have identified that intraoperative blood loss was associated with a higher risk of AKI in cardiac and non‐cardiac surgery [32, 33, 34]. Furthermore, intraoperative blood loss has also been found to be associated with the incidence of AKI in LT [19, 35].

Associations were also observed between higher CONUT scores and several postoperative outcomes, including ventilation time, hospital LOS, EAD incidence, and postoperative MELD scores on POD1, 3, 5, and 7, though these were not subjected to multivariable adjustment. Increased oxidative stress through IL‐1, ‐6 and TNF‐ a [22] has been identified as one of the risk factors for hepatic ischemia/reperfusion injury, which leads to poor graft outcomes, including primary nonfunction, EAD, and higher postoperative MELD scores [36, 37, 38, 39]. Furthermore, previous reports identified that the incidence of EAD was independently associated with longer ventilation time and prolonged hospital LOS [40, 41]. Since EAD and postoperative MELD score are significantly associated with patient and graft survival [17, 42], early prediction of poor outcomes by the preoperative CONUT score can be beneficial. These outcomes were included as exploratory findings to highlight the broader clinical relevance of the CONUT score but were not analyzed as primary endpoints. Further studies are warranted to investigate these associations in more detail with appropriate statistical adjustments.

The present study has several limitations. First, this study was performed retrospectively at a single center. Second, the present study included only living donor LT recipients. Whether our findings can apply to deceased donor LT recipients remains unclear. Third, information regarding perioperative albumin administration, including pretransplant use and postoperative infusion within the first week, was not available in this study. Since albumin supplementation may influence both nutritional status and renal function, the absence of these data limits our ability to account for its potential impact on the observed association between the CONUT score and postoperative AKI. Finally, although there are other widely used nutritional status screening tools, such as the malnutrition universal screening tool or nutrition risk screening‐2002, we did not evaluate them because they include subjective variables. In addition, other objective indices such as the Prognostic Nutritional Index (PNI) and the Nutritional Risk Index (NRI) were not examined in this study. Future research comparing these objective tools may help identify the most appropriate nutritional marker for liver transplant recipients.

In conclusion, this study demonstrated that poor preoperative nutritional status, as assessed by the CONUT score, was independently associated with the incidence of postoperative AKI after living donor liver transplantation. Given its simplicity, objectivity, and availability in routine clinical practice, the CONUT score may serve as a practical adjunct to existing clinical parameters for preoperative risk stratification. Future prospective studies are warranted to validate these findings and to explore whether nutritional optimization prior to liver transplantation can reduce the risk of AKI.

## Author Contributions

Akira Katayama contributed to the research design, data collection, writing of the manuscript, research performance, and data analysis. Ezeldeen Abuelkasem and Marianne M. Ligon contributed to research design, the interpretation of data, and critical review of the manuscript. David W. Wang contributed to research design, the interpretation of data, data analysis, and critical review of the manuscript.

## Conflicts of Interest

The authors declare no conflicts of interest.

## Supporting information



Supplementary information: ctr70562‐sup‐0001‐figureS1.pdf

Supplementary information: ctr70562‐sup‐0002‐figureS2.pdf

Supplementary information: ctr70562‐sup‐0003‐figureS3.pdf

Supplementary information: ctr70562‐sup‐0004‐SuppMat.docx

## Data Availability

The data that support the findings of this study are available from the corresponding author upon reasonable request.
