# Supplementary material for: Controlling Nutritional Status Scores Predict Postoperative Acute Kidney Injury in Living Donor Liver Transplantation
Source: Clin Transplant. 2026 May 14;40:e70562. doi: 10.1111/ctr.70562 (PMC13175226; doi:10.1111/ctr.70562)
Supplement: Supplementary file 1 — Supplementary information: ctr70562‐sup‐0001‐figureS1.pdf [file CTR-40-e70562-s002.pdf]

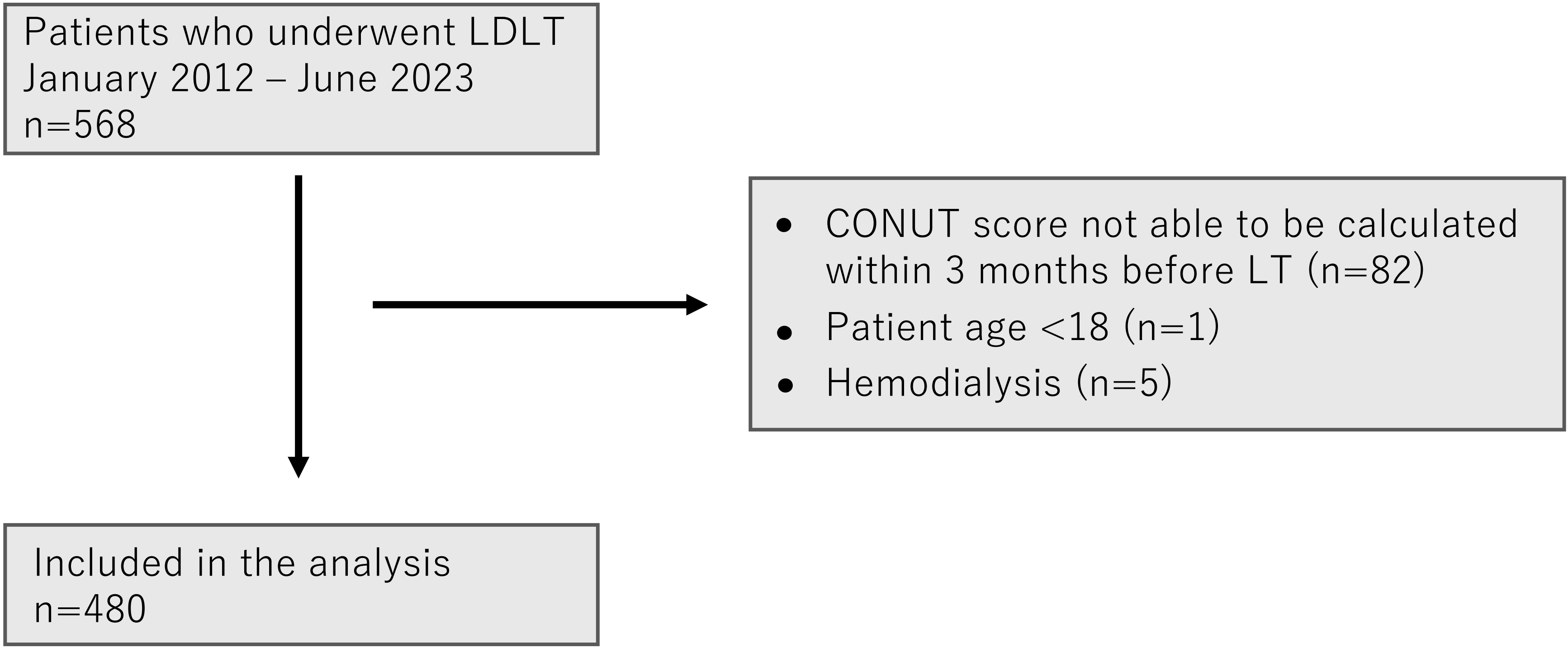

Supplementary Figure 1: Flow diagram of patient selection.

Abbreviation: CONUT, Controlling Nutritional Status; LDLT, living donor liver transplantation; LT, liver transplantation.
