# Supplementary material for: Controlling Nutritional Status Scores Predict Postoperative Acute Kidney Injury in Living Donor Liver Transplantation
Source: Clin Transplant. 2026 May 14;40:e70562. doi: 10.1111/ctr.70562 (PMC13175226; doi:10.1111/ctr.70562)
Supplement: Supplementary file 2 — Supplementary information: ctr70562‐sup‐0002‐figureS2.pdf [file CTR-40-e70562-s004.pdf]

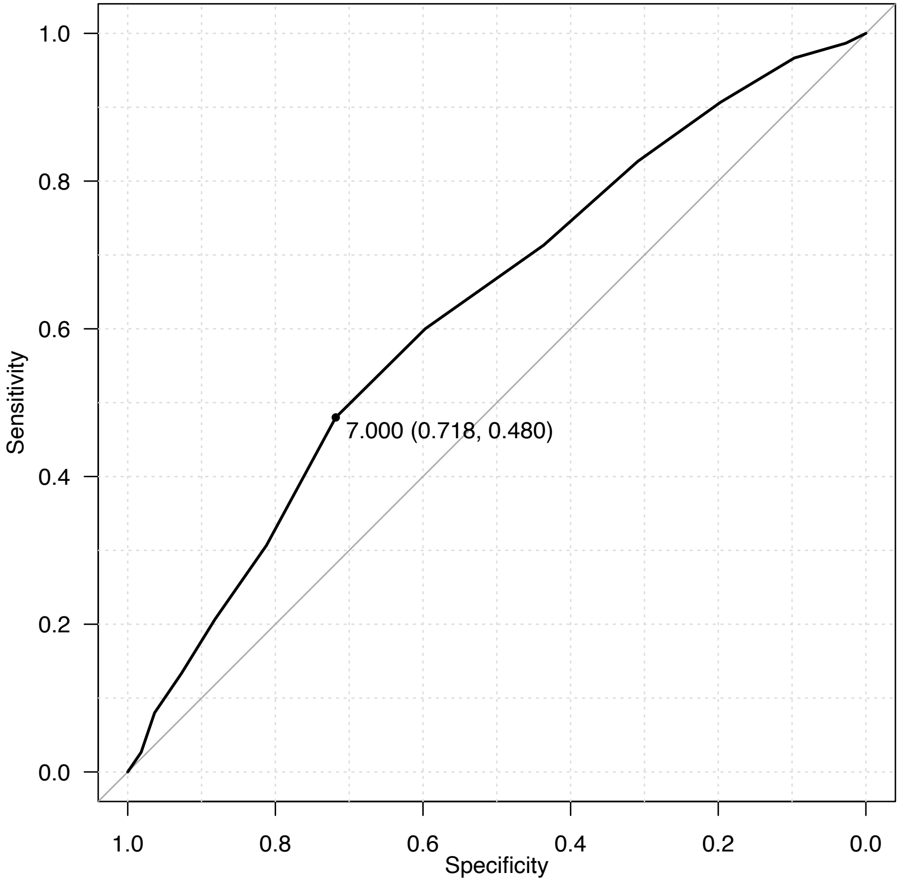


Supplementary Figure 2: ROC curve analysis of the CONUT score for predicting postoperative AKI.

Abbreviations: AKI, acute kidney injury; CONUT, Controlling Nutritional Status.
