# Supplementary material for: Controlling Nutritional Status Scores Predict Postoperative Acute Kidney Injury in Living Donor Liver Transplantation
Source: Clin Transplant. 2026 May 14;40:e70562. doi: 10.1111/ctr.70562 (PMC13175226; doi:10.1111/ctr.70562)
Supplement: Supplementary file 3 — Supplementary information: ctr70562‐sup‐0003‐figureS3.pdf [file CTR-40-e70562-s003.pdf]

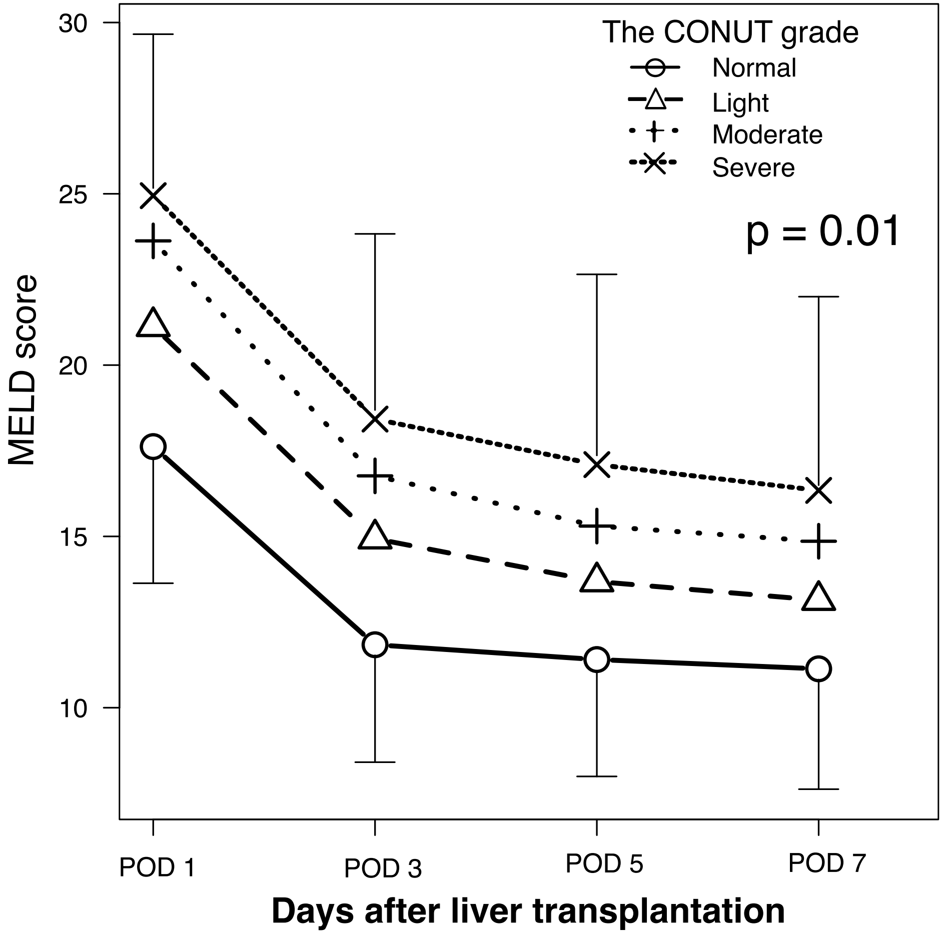

Supplementary Figure 3: Transition of postoperative MELD scores according to CONUT grade.

Abbreviations: CONUT, Controlling Nutritional Status; MELD, model for end-stage liver disease; POD, postoperative day.
