# Supplementary material for: Controlling Nutritional Status Scores Predict Postoperative Acute Kidney Injury in Living Donor Liver Transplantation
Source: Clin Transplant. 2026 May 14;40:e70562. doi: 10.1111/ctr.70562 (PMC13175226; doi:10.1111/ctr.70562)
Supplement: Supplementary file 4 — Supplementary information: ctr70562‐sup‐0004‐SuppMat.docx [file CTR-40-e70562-s001.docx]

Supplementary Figure legends:

Supplementary Figure 1. Flow diagram of patient selection.

A total of 568 patients who underwent living donor liver transplantation between January 2012 and June 2023 were screened. After excluding patients in whom the CONUT score could not be calculated within 3 months before transplantation (n = 82), those aged < 18 years (n = 1), and those receiving hemodialysis (n = 5), 480 patients were included in the final analysis.

Abbreviations: CONUT, Controlling Nutritional Status; LDLT, living donor liver transplantation; LT, liver transplantation.

Supplementary Figure 2. ROC curve analysis of the CONUT score for predicting postoperative AKI.

The area under the curve was 0.62, indicating the discriminatory ability of the CONUT score for AKI. The optimal cutoff value was 7, with a sensitivity of 48.0% and specificity of 71.8%.

Abbreviations: AKI, acute kidney injury; CONUT, Controlling Nutritional Status; ROC, receiver operating curve.

Supplementary Figure 3. Transition of postoperative MELD scores according to CONUT grade.

Postoperative MELD scores were compared across CONUT grade groups (normal, light, moderate, and severe) at postoperative days (POD) 1, 3, 5, and 7. Patients with higher CONUT grades showed persistently elevated MELD scores over time. A significant difference in MELD score among groups was observed (p = 0.01).
